# Awareness of dementia risk factors among healthcare professionals at Hamad Medical Corporation, Qatar: a cross-sectional survey

**DOI:** 10.3389/fpubh.2024.1443525

**Published:** 2024-09-30

**Authors:** Hanadi Al Hamad, Brijesh Sathian

**Affiliations:** ^1^Department of Geriatrics and Long-Term Care, Rumailah Hospital, Hamad Medical Corporation, Doha, Qatar; ^2^Qatar Rehabilitation Institute, Hamad Medical Corporation, Doha, Qatar; ^3^WHO Collaborating Centre for Healthy Ageing and Dementia, Doha, Qatar

**Keywords:** dementia risk reduction, risk factors, healthcare professionals, awareness, Qatar

## Abstract

**Background:**

Dementia, a degenerative neurological disorder, is estimated to affect 82 million people worldwide by 2030 and 152 million by 2050, with a sharp increase in its incidence in the Middle East and Qatar. Lifestyle factors, such as smoking, physical inactivity, and obesity, may account for up to 40% of dementia cases. Healthcare practitioners who play an important role in health promotion must understand the modifiable risk and protective factors for dementia. This study investigated healthcare professionals' knowledge of dementia risk factors at Hamad Medical Corporation in Qatar.

**Methods:**

A sample of 737 healthcare professionals was recruited using simple random sampling from Hamad Medical Corporation. The target population included physicians, nurses, and allied healthcare workers from various departments. The participants completed an online survey between 1st January and December 31, 2023. The survey included questions on modifiable risk factors, preventive interventions, and dementia-related information sources.

**Results:**

The study revealed that 76% of participants believed in dementia prevention and 87.4% were interested in learning about lifestyle choices and dementia risk. The majority of the participants were female and non-Qataris. Challenges to dementia risk reduction include lack of understanding, time restrictions, and motivational obstacles. Awareness of risk factors such as depression, alcohol use, and physical inactivity was identified. Digital platforms are the dominant source of information, highlighting the need for more dementia education and prevention programs.

**Conclusion:**

Given the gaps identified in knowledge, we recommend further training to improve the knowledge of healthcare professionals. In addition, further exploration of patients and caregiverss is warranted.

## 1 Introduction

Dementia is a progressive neurological condition that causes memory loss, difficulties with daily tasks, and personality changes (1). As people live longer, the global burden of dementia increases. The increasing number of people with dementia is associated with higher healthcare expenses and greater strain on caregivers, families, and society (2). The World Health Organization (WHO) predicts that dementia will affect 82 million individuals by 2030 and 152 million by 2050 (1). The prevalence of dementia in the Middle East and Qatar is expected to increase dramatically by 2050 (3). Dementia research has recently shifted to modifiable factors related to lifestyle, such as smoking, physical inactivity, and obesity, which may account for up to 40% of dementia cases globally (4). These findings support early prevention through lifestyle measures, as recommended by the WHO on cognitive decline and dementia risk reduction (1). As the aging population expands exponentially, the public needs to understand modifiable risk and protective factors for dementia. Healthcare professionals who play a pivotal role in health promotion must have knowledge of preventative strategies (5). De Krom et al reported that 75% of the health care professionals knows about dementia risk reduction (5).

This study aimed to analyze healthcare workers' knowledge of dementia risk factors at Hamad Medical Corporation (HMC), Qatar's leading healthcare provider. Understanding the existing level of knowledge among healthcare workers allows us to identify areas for focused educational interventions and to improve Qatar's capacity to reduce the burden of dementia.

## 2 Methods

### 2.1 Study design and participants

#### 2.1.1 Study design

This study used a prospective survey methodology to measure healthcare workers' knowledge and awareness of dementia risk factors at Hamad Medical Corporation (HMC) in Qatar. HMC is Qatar's largest secondary and tertiary healthcare provider, operating 12 hospitals, nine specialized and three community hospitals, the National Ambulance Service, and home and residential care services.

#### 2.1.2 Study population and setting

The target population consisted of healthcare professionals from various departments and specializations at HMC, including doctors, nurses, and allied health workers.

#### 2.1.3 Sampling

The sampling frame included five major hospitals that have older adults care services: Rumailah Hospital, Hamad General Hospital, Al Wakrah Hospital, Al Khor Hospital, and Heart Hospital. A sample size of 750 healthcare professionals was needed for the survey, based on an expected knowledge of 50% of dementia risk factors among healthcare workers and a significance level of 5%. We planned to screen 850 participants considering their lack of response. A total of 170 e-mail addresses were randomly selected from each hospital to obtain a list of 850 participants. We obtained 737 responses with an overall response rate of 86%. The hospital-wise breakdown was Al Khor Hospital (105), Al Wakrah Hospital (167), Hamad General Hospital (160), Heart Hospital (146), and Rumailah Hospital (159). Among the five hospitals, the Geriatric Memory Clinic is only in Rumailah Hospital, with a staff structure of 30% male and 9% Qataris. Participants were invited to complete an online survey between January 1, 2023, and December 31, 2023.

#### 2.1.4 Data collection

A research assistant contacted the healthcare providers face to face and handed them a research information leaflet and an iPad carrying an electronic questionnaire. The survey, which took ~6–8 min to complete, comprised questions that tested the participants' knowledge of dementia risk factors and preventative methods. Participants provided anonymous replies directly on the iPad.

### 2.2 Outcome measures

The survey instrument contained items that addressed several elements of dementia risk factors, such as awareness of modifiable risk factors (e.g., hypertension, diabetes, and smoking), knowledge of preventative interventions, and use of dementia-related information sources (4). Demographic data, including occupation and practice settings, were also collected.

### 2.3 Statistical analysis

Descriptive statistics were used to summarize the participants' demographic information and survey responses. The level of knowledge of dementia risk factors was determined by analyzing participants' responses to pertinent survey questions. Subgroup analyses were used to investigate the differences in awareness levels across professional and practice settings. Statistical significance was determined using the chi-square test, with a *p* < 0.05. All statistical tests were performed using the R-4.3.2 for Windows software.

## 3 Results

In this study of healthcare professionals in Qatar, a significant number (76%) believed in dementia prevention, indicating a hopeful attitude toward preventive brain health treatments. Furthermore, a sizable majority (87.4%) expressed interest in learning more about the link between lifestyle choices and dementia risk (Table 1). The gender distribution was biased toward females (58.5%), with non-Qataris being the vast majority of respondents (95.7%). The Geriatric Memory Clinic at HMC was quite well-known (50.7%), highlighting attempts to increase institutional awareness. However, challenges to dementia management remain, with a large proportion noting lack of understanding (76.5%), time restrictions (54.0%), and motivational obstacles (60.8%). Depression (69.1%), excessive alcohol use (74.8%), and physical inactivity (65.5%) were among the most frequently recognized risk factors. Digital platforms, specifically the Internet (70.0%), have emerged as the dominant source of information, indicating a dependence on technologically driven resources. These findings highlight both positive views on dementia prevention and persistent issues in knowledge dissemination and resource accessibility within Qatar's healthcare community.

**Table 1:** Gender wise cross tabulation of knowledge and other factors.

**Questions**	**Gender, number (%)**		***P*** **value**	
	**Female**	**Male**	**Total**	
Do you think dementia can be prevented?				0.255
No	97 (22.5%)	80 (26.1%)	177 (24%)	
Yes	334 (77.5%)	226 (73.9%)	560 (76%)	
Would you like to receive more information about the relationship between lifestyle and brain health/dementia risk?				0.003
No	41 (9.5%)	52 (17/0%)	93 (12.6%)	
Yes	390 (90.5%)	254 (83.0%)	644 (87.4%)	
By modifying all risk factors, what percent of dementia can be prevented or delayed?				0.068
20	84 (19.5%)	82 (26.8%)	166 (22.5%)	
40	177 (41.1%)	126 (41.2%)	303 (41.1%)	
50	124 (28.8%)	69 (22.5%)	193 (26.2%)	
60	46 (10.7%)	29 (9.5%)	75 (10.2%)	
Are you aware about the Geriatric Memory Clinic at HMC?				0.966
No	212 (49.2%)	151 (49.3%)	363 (49.3%)	
Yes	219 (50.8%)	155 (50.7%)	374 (50.7%)	
**What are the largest barriers for implementing a brain-healthy lifestyle in daily life?**				
Lack of knowledge				0.037
No	113 (26.2%)	60 (19.6%)	173 (23.5%)	
Yes	318 (73.8%)	246 (80.4%)	564 (76.5%)	
Lack of time				0.680
No	201 (46.6%)	138 (45.1%)	339 (46.0%)	
Yes	230 (53.4%)	168 (54.9%)	398 (54.0%)	
Lack of motivation				0.645
No	166 (38.5%)	123 (40.2%)	289 (39.2%)	
Yes	265 (61.5%)	183 (59.8%)	448 (60.8%)	
Financial reasons				0.526
No	302 (70.1%)	221 (72.2%)	523 (71.0%)	
Yes	129 (29.9%)	85 (27.8%)	214 (29.0%)	
Difficulties with organizing				0.428
No	268 (62.2%)	199 (65.0%)	467 (63.4%)	
Yes	163 (37.8%)	107 (35.0%)	270 (36.6%)	
Health condition				<0.001
No	203 (47.1%)	186 (60.8%)	389 (52.8%)	
Yes	228 (52.9%)	120 (39.2%)	348 (47.2%)	
**Please identify the “modifiable risk factors” for dementia from the below**				
Hypertension				0.004
No	238 (55.2%)	136 (44.4%)	374 (50.7%)	
Yes	193 (44.8%)	170 (55.6%)	363 (49.3%)	
Depression				0.118
No	143 (33.2%)	85 (27.8%)	228 (30.9%)	
Yes	288 (66.8%)	221 (72.2%)	509 (69.1%)	
Excessive alcohol consumption				<0.001
No	132 (30.6%)	54 (17.6%)	186 (25.2%)	
Yes	299 (69.4%)	252 (82.4%)	551 (74.8%)	
Less education				0.575
No	293 (68.0%)	202 (66.0%)	495 (67.2%)	
Yes	138 (32.0%)	104 (34.0%)	242 (32.8%)	
Smoking				<0.001
No	180 (41.8%)	72 (23.5%)	252 (34.2%)	
Yes	251 (58.2%)	234 (76.5%)	485 (65.8%)	
Head injury				0.166
No	211 (49.0%)	134 (43.8%)	345 (46.8%)	
Yes	220 (51.0%)	172 (56.2%)	392 (53.2%)	
Air pollution				0.249
No	314 (72.9%)	211 (69.0%)	525 (71.2%)	
Yes	117 (27.1%)	95 (31.0%)	212 (28.8%)	
Infrequent social contact				0.181
No	230 (53.4%)	148 (48.4%)	378 (51.3%)	
Yes	201 (46.6%)	158 (51.6%)	359 (48.7%)	
Diabetes				0.005
No	240 (55.7%)	138 (45.1%)	378 (51.3%)	
Yes	191 (44.3%)	168 (54.9%)	359 (48.7%)	
Obesity				0.005
No	226 (52.4%)	128 (41.8%)	354 (48.0%)	
Yes	205 (47.6%)	178 (58.2%)	383 (52.0%)	
Stroke				0.002
No	248 (57.5%)	141 (46.1%)	389 (52.8%)	
Yes	183 (42.5%)	165 (53.9%)	348 (47.2%)	
Hearing impairment				<0.001
No	336 (78.0%)	202 (66.0%)	538 (73.0%)	
Yes	95 (22.0%)	104 (34.0%)	199 (27.0%)	
Physical inactivity				0.183
No	157 (36.4%)	97 (31.7%)	254 (34.5%)	
Yes	274 (63.6%)	209 (68.3%)	483 (65.5%)	
**From where you would like to get the information regarding dementia risk factors?**				
Alzheimer's disease associations				0.992
No	321 (74.5%)	228 (74.5%)	549 (74.5%)	
Yes	110 (25.5%)	78 (25.5%)	188 (25.5%)	
Internet				0.270
No	136 (31.6%)	85 (27.8%)	221 (30.0%)	
Yes	295 (68.4%)	221 (72.2%)	516 (70.0%)	
Healthy aging website HMC				0.340
No	244 (56.6%)	184 (60.1%)	428 (58.1%)	
Yes	187 (43.4%)	122 (39.9%)	309 (41.9%)	
Memory clinic training program HMC				0.281
No	242 (56.1%)	184 (60.1%)	426 (57.8%)	
Yes	189 (43.9%)	122 (39.9%)	311 (42.2%)	

Table 1 shows that 77.5% of females and 73.9% of males believed that dementia was preventable (p = 0.255). Females (90.5%) were substantially more interested in learning about the association between lifestyle and brain health/dementia risk than males (83.0%) (*p* = 0.003). While beliefs about the percentage of dementia that may be prevented or postponed by risk factor adjustment differed, there were no statistically significant sex differences (*p* = 0.068). Awareness of HMC's Geriatric Memory Clinic was consistent across genders (*p* = 0.966). Barriers to dementia treatment, such as lack of understanding, revealed substantial gender differences, with 73.8% of females and 80.4% of males reporting this hurdle. Females had a higher awareness of risk factors such as hypertension (*p* = 0.004), depression (*p* = 0.118), excessive alcohol consumption (*p* < 0.001), smoking (*p* < 0.001), diabetes (*p* = 0.005), obesity (*p* = 0.005), stroke (*p* = 0.002), and hearing impairment (*p* < 0.001). In contrast, men showed higher levels of awareness of head injury (*p* = 0.166). There were no significant sex disparities across dementia information sources.

Supplementary Table 1 shows the cross-tabulation results, which examined the relationship between years of experience and replies to several dementia-related questions. There was no significant difference in opinions regarding how to prevent dementia between healthcare workers with more than 10 years of experience and those with fewer (*p* = 0.399). Similarly, the need for further knowledge regarding lifestyle and brain health/dementia risk did not differ substantially according to years of experience (*p* = 0.745). However, there was a statistically significant difference in the assessment of the proportion of dementia that may be prevented or delayed by modifying the risk factors (*p* = 0.018). There was a substantial difference in the knowledge of the Geriatric Memory Clinic at HMC based on years of experience, with more experienced individuals having higher awareness (*p* < 0.001). Furthermore, while a lack of motivation to address dementia showed a significant difference (*p* = 0.003), other barriers such as lack of knowledge, time constraints, financial reasons, difficulties with organization, and health conditions did not show a significant relationship with years of experience. Except for air pollution, years of experience had no significant effect on reactions to the individual risk variables (*p* = 0.046). Similarly, sources of dementia knowledge did not differ significantly based on years of experience, with the exception of HMC's memory clinic training program (*p* = 0.039). These data indicate that whereas certain components of dementia knowledge and perception may be altered by years of experience, others remain similar among healthcare workers regardless of tenure.

In Supplementary Table 2, we present cross-tabulation data that investigate the relationship between profession and responses to numerous dementia-related questions. There was no significant difference in beliefs about the preventability of dementia among the occupations (*p* = 0.960). However, there was a significant difference in the need for further knowledge concerning the link between lifestyle and brain health/dementia risk (*p* = 0.006), with physicians showing less interest than did allied health professionals and nurses. There were significant differences in the perceptions of the percentage of dementia avoidable or postponed with risk factor adjustment (*p* < 0.001), with doctors being less hopeful than allied health workers and nurses. There was no significant difference in awareness of HMC geriatric memory clinics by occupation (*p* = 0.381). Barriers to dementia treatment differed by occupation, with significant variations in time constraints (*p* = 0.018) and health problems (*p* < 0.001). Significant differences in the risk variables for dementia were found across occupations, including hypertension, excessive alcohol use, low education, smoking, air pollution, infrequent social interaction, diabetes, obesity, stroke, and hearing impairment (all *p* < 0.001). Sources of dementia information revealed varying relationships with career, with significant variations reported for the Internet (*p* = 0.011) and the HMC Healthy Aging Website (*p* = 0.014). There were no significant differences between awareness of Alzheimer's disease linkages and HMC's memory clinic training program. These findings highlight the necessity of considering professional responsibilities and viewpoints when designing dementia therapies and educational programs.

## 4 Discussion

This study highlights an urgent requirement for specific educational interventions aimed at enhancing knowledge about dementia risk factors among healthcare personnel at the Hamad Medical Corporation (HMC) in Qatar. We evaluated the participants' views of possibilities for preventing dementia and reducing risk, as well as their levels of awareness on specific risk factors. De Krom et al observed that 75% of the current healthcare staff knew about dementia risk factors, which is consistent with our findings (5). Despite extensive evidence that lifestyle modifications can considerably lower dementia risk, many participants in our study were unaware of the preventative benefits of treating risk factors, such as hearing loss, hypertension, diabetes, physical inactivity, and smoking (4). Another study conducted in Queensland, Australia, discovered that dementia knowledge among healthcare district workers was modest, with substantial variances depending on professional groups and experience caring for dementia patients (6). Certain domains, notably those related to medicine, had lower levels of knowledge. Those who participated in dementia-specific training, such as attending relevant seminars, had greater levels of knowledge. These findings indicate that dementia-specific training may increase understanding and promote the implementation of systematic dementia-specific education or training.

Four studies from the Middle East reported findings regarding awareness of dementia (7–10). Paul et al. conducted a study to determine healthcare stakeholders' knowledge, awareness, and attitudes regarding Alzheimer's disease and dementia in Qatar. They discovered that more than 70% of 229 participants had not received related education or training in the previous 2 years. Healthcare workers had an intermediate understanding of dementia and Alzheimer's disease, but they were unaware of recent developments in fundamental disease pathophysiology. There were differences in respondents' occupations and locations of respondents (7). Werner et al. found that while the majority of family physicians in one of Israel's largest Health Maintenance Organizations (HMOs) had heard of MCI, a third of those who were aware with the term knew essentially nothing about it (8). Participants had considerable objective knowledge of the numerous causes of MCI, yet 70% believed it was caused by natural aging. Help-seeking and treatment choices were consistent with the literature on MCI. Shinan-Altman et al. surveyed 327 Israeli nurses and social workers to report their cognitive and emotional representations (9). Knowledge of Alzheimer's, demographics, and employment factors was also acquired. The participants perceived AD as a chronic condition with serious effects. There were statistically significant differences between the groups, with nurses attributing more psychological factors to AD than social workers did. Nonetheless, social workers considered Alzheimer's be more chronic and with serious repercussions than nurses believed. Despite these similarities, there were discrepancies in how social workers and nurses represented AD disease. According to Ayalon et al., more than 30% of Israel's home care workers disagreed with scientific views on Alzheimer's disease and associated dementia (ADRD) (10). Workers who were not told about the care recipient's medical issues were more likely to express attitudes that contradicted their current scientific understanding. In qualitative interviews, they described employing natural behavioral strategies to care for older adults with ADRD.

Our study found disparities in awareness levels across occupational groups. Nurses and allied health workers were more aware of lifestyle risk factors than physicians, which is contrary to the findings of other studies (11–14). This emphasizes the importance of tailoring training curricula to satisfy the educational needs of different professional groups. Internet was a major source of information, notably among physicians. Using a variety of outlets such as professional organizations, educational seminars, and online resources can improve the reach and efficacy of educational initiatives. Several dementia education programs and tools have been evaluated for their usefulness in increasing dementia awareness (15–17). All studies revealed that these programs improved healthcare workers' attitudes about caring for patients with dementia, as well as their overall knowledge. To promote exceptional dementia care, health services should strive for a high number of workers who have received dementia-specific training. This may need to make dementia education an essential part of employment standards and staff development programs.

## 5 Strengths and limitations

The strength of this study is that it provides a thorough understanding of healthcare professionals' knowledge regarding the dementia risk reduction and high response rate. The study's shortcomings include the fewer sample size for few subgroups analysis which include nationality and practice setting. We have not considered same hospital how many to get from the practice setting such as acute vs. long term care. we were not able to collect the respective fields of the health care professional. To manage social desirability bias, the data were collected using an electronic device (iPad using Microsoft forms) that was anonymized and de-identified. This ensured that participants provided accurate and honest responses. Furthermore, the study's cross-sectional approach precludes the detection of causal relationships between the variables.

## 6 Conclusion

This study emphasizes the need for dementia-specific training and education to enhance public healthcare for persons with dementia. This illustrates the need to identify risk factors for Alzheimer's disease and how they progress to provide optimal dementia care. Employers can oversee and encourage education or training in this field through health degree programs.

Dementia-specific education might significantly contribute to care transformation because it empowers professionals with the necessary skill sets to offer high-quality care to older adults. Further studies are needed to determine whether various types or facets of dementia education or training are associated with improved attitudes and confidence in caring for patients with dementia. This study adds to the expanding body of research on dementia care delivery and offers useful insights to policymakers, healthcare professionals, and other stakeholders involved in dementia prevention and treatment. Prioritizing innovative strategies for risk reduction and health promotion is necessary.

## Data Availability

The original contributions presented in the study are included in the article/Supplementary material, further inquiries can be directed to the corresponding author.
