# Supplementary material for: Awareness of dementia risk factors among healthcare professionals at Hamad Medical Corporation, Qatar: a cross-sectional survey
Source: Front Public Health. 2024 Sep 30;12:1443525. doi: 10.3389/fpubh.2024.1443525 (PMC11471612; doi:10.3389/fpubh.2024.1443525)
Supplement: Supplementary file 1 [file Table_1.DOCX]

**Supplementary 1. Years of Experience wise crosstabulation of knowledge and other factors**

| **QUESTIONS** | **Years of Experience, Number (%)** | | **P Value** |
| --- | --- | --- | --- |
|  | **≥10 Years** | **<10 Years** |  |
| Do you think dementia can be prevented? |  |  | 0.399 |
| No | 127 (24.9%) | 50 (22.0%) |  |
| Yes | 383 (75.1%) | 177 (78.0%) |  |
| Would you like to receive more information about the relationship between lifestyle and brain health/dementia risk? |  |  | 0.745 |
| No | 63 (12.4%) | 30 (13.2%) |  |
| Yes | 447 (87.6%) | 197 (86.8%) |  |
| By modifying all risk factors, what percent of dementia can be prevented or delayed? |  |  | 0.018 |
| 20 | 105 (20.6%) | 61 (26.9%) |  |
| 40 | 201 (39.4%) | 102 (44.9%) |  |
| 50 | 146 (28.6%) | 47 (20.7%) |  |
| 60 | 58 (11.4%) | 17 (7.5%) |  |
| Are you aware about the Geriatric Memory Clinic at HMC? |  |  | <0.001 |
| No | 225 (44.1%) | 138 (60.8%) |  |
| Yes | 285 (55.9%) | 89 (39.2%) |  |
| What are the largest barriers for implementing a brain-healthy lifestyle in daily life? |  |  |  |
| Lack of knowledge |  |  | 0.536 |
| No | 123 (24.1%) | 50 (22.0%) |  |
| Yes | 387 (75.9%) | 177 (78.0%) |  |
| Lack of time |  |  | 0.800 |
| No | 233 (45.7%) | 106 (46.7%) |  |
| Yes | 277 (54.3%) | 121 (53.3%) |  |
| Lack of motivation |  |  | 0.003 |
| No | 182 (35.7%) | 107 (47.1%) |  |
| Yes | 328 (64.3%) | 120 (52.9%) |  |
| Financial reasons |  |  | 0.848 |
| No | 363 (71.2%) | 160 (70.5%) |  |
| Yes | 147 (28.8%) | 67 (29.5%) |  |
| Difficulties with organizing |  |  | 0.236 |
| No | 316 (62.0%) | 151 (66.5%) |  |
| Yes | 194 (38.0%) | 76 (33.5%) |  |
| Health condition |  |  | 0.007 |
| No | 286 (56.1%) | 103 (45.4%) |  |
| Yes | 224 (43.9%) | 124 (54.6%) |  |
| Please identify the ‘modifiable risk factors’ for dementia from the below |  |  |  |
| Hypertension |  |  | 0.277 |
| No | 252 (49.4%) | 122 (53.7%) |  |
| Yes | 258 (50.6%) | 105 (46.3%) |  |
| Depression |  |  | 0.180 |
| No | 150 (29.4%) | 78 (34.4%) |  |
| Yes | 360 (70.6%) | 149 (65.6%) |  |
| Excessive alcohol consumption |  |  | 0.110 |
| No | 120 (23.5%) | 66 (29.1%) |  |
| Yes | 390 (76.5%) | 161 (70.9%) |  |
| Less education |  |  | 0.804 |
| No | 344 (67.5%) | 151 (66.5%) |  |
| Yes | 166 (32.5%) | 76 (33.5%) |  |
| Smoking |  |  | 0.917 |
| No | 175 (34.3%) | 77 (33.9%) |  |
| Yes | 335 (65.7%) | 150 (66.1%) |  |
| Head injury |  |  | 0.906 |
| No | 238 (46.7%) | 107 (47.1%) |  |
| Yes | 272 (53.3%) | 120 (52.9%) |  |
| Air pollution |  |  | 0.046 |
| No | 352 (69.0%) | 173 (76.2%) |  |
| Yes | 158 (31.0%) | 54 (23.8%) |  |
| Infrequent social contact |  |  | 0.568 |
| No | 258 (50.6%) | 120 (52.9%) |  |
| Yes | 252 (49.4%) | 107 (47.1%) |  |
| Diabetes |  |  | 0.227 |
| No | 254 (49.8%) | 124 (54.6%) |  |
| Yes | 256 (50.2%) | 103 (45.4%) |  |
| Obesity |  |  | 0.341 |
| No | 239 (46.9%) | 115 (50.7%) |  |
| Yes | 271 (53.1%) | 112 (49.3%) |  |
| Stroke |  |  | 0.142 |
| No | 260 (51.0%) | 129 (56.8%) |  |
| Yes | 250 (49.0%) | 98 (43.2%) |  |
| Hearing impairment |  |  | 0.341 |
| No | 367 (72.0%) | 171 (75.3%) |  |
| Yes | 143 (28.0%) | 56 (24.7%) |  |
| Physical inactivity |  |  | 0.424 |
| No | 171 (33.5%) | 83 (36.6%) |  |
| Yes | 339 (66.5%) | 144 (63.4%) |  |
| From where you would like to get the information regarding dementia risk factors? |  |  |  |
| Alzheimer’s disease associations |  |  | 0.868 |
| No | 379 (74.3%) | 170 (74.9%) |  |
| Yes | 131 (25.7%) | 57 (25.1%) |  |
| Internet |  |  | 0.593 |
| No | 156 (30.6%) | 65 (28.6%) |  |
| Yes | 354 (69.4%) | 162 (71.4%) |  |
| Healthy Aging website HMC |  |  | 0.138 |
| No | 287 (56.3%) | 141 (62.1%) |  |
| Yes | 223 (43.7%) | 86 (37.9%) |  |
| Memory clinic training program HMC |  |  | 0.039 |
| No | 282 (55.3%) | 144 (63.4%) |  |
| Yes | 228 (44.7%) | 83 (36.6%) |  |

**Supplementary Table 2. Profession wise crosstabulation of knowledge and other factors**

| **QUESTIONS** | **Profession, Number (%)** | | | **P Value** |
| --- | --- | --- | --- | --- |
|  | **Allied Health** | **Nurse** | **Physician** |  |
| Do you think dementia can be prevented? |  |  |  | 0.960 |
| No | 62 (24.5%) | 65 (23.5%) | 50 (24.2%) |  |
| Yes | 191 (75.5%) | 212 (76.5%) | 157 (75.8%) |  |
| Would you like to receive more information about the relationship between lifestyle and brain health/dementia risk? |  |  |  | 0.006 |
| No | 31 (12.3%) | 24 (8.7%) | 38 (18.4%) |  |
| Yes | 222 (87.7%) | 253 (91.3%) | 169 (81.6%) |  |
| By modifying all risk factors, what percent of dementia can be prevented or delayed? |  |  |  | <0.001 |
| 20 | 50 (19.8%) | 37 (13.4%) | 79 (38.2%) |  |
| 40 | 97 (38.3%) | 122 (44.0%) | 84 (40.6%) |  |
| 50 | 79 (31.2%) | 85 (30.7%) | 29 (14.0%) |  |
| 60 | 27 (10.7%) | 33 (11.9%) | 15 (7.2%) |  |
| Are you aware about the Geriatric Memory Clinic at HMC? |  |  |  | 0.381 |
| No | 133 (52.6%) | 129 (46.6%) | 101 (48.8%) |  |
| Yes | 120 (47.4%) | 148 (53.4%) | 106 (51.2%) |  |
| What are the largest barriers for implementing a brain-healthy lifestyle in daily life? |  |  |  |  |
| Lack of knowledge |  |  |  | 0.646 |
| No | 63 (24.9%) | 66 (23.8%) | 44 (21.3%) |  |
| Yes | 190 (75.1%) | 211 (76.2%) | 163 (78.7%) |  |
| Lack of time |  |  |  | 0.018 |
| No | 134 (53.0%) | 121 (43.7%) | 84 (40.6%) |  |
| Yes | 119 (47.0%) | 156 (56.3%) | 123 (59.4%) |  |
| Lack of motivation |  |  |  | 0.052 |
| No | 108 (42.7%) | 93 (33.6%) | 88 (42.5%) |  |
| Yes | 145 (57.3%) | 184 (66.4%) | 119 (57.5%) |  |
| Financial reasons |  |  |  | 0.196 |
| No | 169 (66.8%) | 202 (72.9%) | 152 (73.4%) |  |
| Yes | 84 (33.2%) | 75 (27.1%) | 55 (26.6%) |  |
| Difficulties with organizing |  |  |  | 0.326 |
| No | 155 (61.3%) | 185 (66.8%) | 127 (61.4%) |  |
| Yes | 98 (38.7%) | 92 (33.2%) | 80 (38.6%) |  |
| Health condition |  |  |  | <0.001 |
| No | 127 (50.2%) | 112 (40.4%) | 150 (72.5%) |  |
| Yes | 126 (49.8%) | 165 (59.6%) | 57 (27.5%) |  |
| Please identify the ‘modifiable risk factors’ for dementia from the below |  |  |  |  |
| Hypertension |  |  |  | <0.001 |
| No | 125 (49.4%) | 174 (62.8%) | 75 (36.2%) |  |
| Yes | 128 (50.6%) | 103 (37.2%) | 132 (63.8%) |  |
| Depression |  |  |  | 0.033 |
| No | 76 (30.0%) | 100 (36.1%) | 52 (25.1%) |  |
| Yes | 177 (70.0%) | 177 (63.9%) | 155 (74.9%) |  |
| Excessive alcohol consumption |  |  |  | <0.001 |
| No | 70 (27.7%) | 90 (32.5%) | 26 (12.6%) |  |
| Yes | 183 (72.3%) | 187 (67.5%) | 181 (87.4%) |  |
| Less education |  |  |  | 0.007 |
| No | 163 (64.4%) | 205 (74.0%) | 127 (61.4%) |  |
| Yes | 90 (35.6%) | 72 (26.0%) | 80 (38.6%) |  |
| Smoking |  |  |  | <0.001 |
| No | 87 (34.4%) | 131 (47.3%) | 34 (16.4%) |  |
| Yes | 166 (65.6%) | 146 (52.7%) | 173 (83.6%) |  |
| Head injury |  |  |  | 0.106 |
| No | 125 (49.4%) | 136 (49.1%) | 84 (40.6%) |  |
| Yes | 128 (50.6%) | 141 (50.9%) | 123 (59.4%) |  |
| Air pollution |  |  |  | <0.001 |
| No | 185 (73.1%) | 217 (78.3%) | 123 (59.4%) |  |
| Yes | 68 (26.9%) | 60 (21.7%) | 84 (40.6%) |  |
| Infrequent social contact |  |  |  | <0.001 |
| No | 129 (51.0%) | 175 (63.2%) | 74 (35.7%) |  |
| Yes | 124 (49.0%) | 102 (36.8%) | 133 (64.3%) |  |
| Diabetes |  |  |  | <0.001 |
| No | 146 (57.7%) | 168 (60.6%) | 64 (30.9%) |  |
| Yes | 107 (42.3%) | 109 (39.4%) | 143 (69.1%) |  |
| Obesity |  |  |  | <0.001 |
| No | 112 (44.3%) | 160 (57.8%) | 82 (39.6%) |  |
| Yes | 141 (55.7%) | 117 (42.2%) | 125 (60.4%) |  |
| Stroke |  |  |  | <0.001 |
| No | 149 (58.9%) | 154 (55.6%) | 86 (41.5%) |  |
| Yes | 104 (41.1%) | 123 (44.4%) | 121 (58.5%) |  |
| Hearing impairment |  |  |  | <0.001 |
| No | 202 (79.8%) | 222 (80.1%) | 114 (55.1%) |  |
| Yes | 51 (20.2%) | 55 (19.9%) | 93 (44.9%) |  |
| Physical inactivity |  |  |  | 0.005 |
| No | 86 (34.0%) | 113 (40.8%) | 55 (26.6%) |  |
| Yes | 167 (66.0%) | 164 (59.2%) | 152 (73.4%) |  |
| From where you would like to get the information regarding dementia risk factors? |  |  |  |  |
| Alzheimer’s disease associations |  |  |  | 0.577 |
| No | 187 (73.9%) | 212 (76.5%) | 150 (72.5%) |  |
| Yes | 66 (26.1%) | 65 (23.5%) | 57 (27.5%) |  |
| Internet |  |  |  | 0.011 |
| No | 85 (33.6%) | 65 (23.5%) | 71 (34.3%) |  |
| Yes | 168 (66.4%) | 212 (76.5%) | 136 (65.7%) |  |
| Healthy Aging website HMC |  |  |  | 0.014 |
| No | 131 (51.8%) | 162 (58.5%) | 135 (65.2%) |  |
| Yes | 122 (48.2%) | 115 (41.5%) | 72 (34.8%) |  |
| Memory clinic training program HMC |  |  |  | 0.822 |
| No | 143 (56.5%) | 160 (57.8%) | 123 (59.4%) |  |
| Yes | 110 (43.5%) | 117 (42.2%) | 84 (40.6%) |  |

**Supplementary Table 3. Hospital wise crosstabulation of knowledge and other factors**

| **QUESTIONS** | **Hospital, Number (%)** | | | | | **P Value** |
| --- | --- | --- | --- | --- | --- | --- |
|  | **Al Khor** | **Al Wakra** | **Hamad General Hospital** | **Heart Hospital** | **Rumailah Hospital** |  |
| Do you think dementia can be prevented? |  |  |  |  |  | 0.329 |
| No | 27 (25.7%) | 44 (26.3%) | 37 (23.1%) | 26 (17.8%) | 43 (27.0%) |  |
| Yes | 78 (74.3%) | 123 (73.7%) | 123 (76.9%) | 120 (82.2%) | 116 (73.0%) |  |
| Would you like to receive more information about the relationship between lifestyle and brain health/dementia risk? |  |  |  |  |  | 0.004 |
| No | 11 (10.5%) | 16 (9.6%) | 33 (20.6%) | 21 (14.4%) | 12 (7.5%) |  |
| Yes | 94 (89.5%) | 151 (90.4%) | 127 (79.4%) | 125 (85.6%) | 147 (87.4%) |  |
| By modifying all risk factors, what percent of dementia can be prevented or delayed? |  |  |  |  |  | 0.034 |
| 20 | 19 (18.1%) | 41 (24.6%) | 48 (30.0%) | 33 (22.6%) | 25 (15.7%) |  |
| 40 | 43 (41.0%) | 63 (37.7%) | 63 (39.4%) | 66 (45.2%) | 68 (42.8%) |  |
| 50 | 29 (27.6%) | 44 (26.3%) | 35 (21.9%) | 42 (28.8%) | 43 (27.0%) |  |
| 60 | 14 (13.3%) | 19 (11.4%) | 14 (8.8%) | 5 (3.4%) | 23 (14.5%) |  |
| Are you aware about the Geriatric Memory Clinic at HMC? |  |  |  |  |  | <.001 |
| No | 58 (55.2%) | 82 (49.1%) | 107 (66.9%) | 93 (63.7%) | 23 (14.5%) |  |
| Yes | 47 (44.8%) | 85 (50.9%) | 53 (33.1%) | 53 (36.3%) | 136 (85.5%) |  |
| What are the largest barriers for implementing a brain-healthy lifestyle in daily life? |  |  |  |  |  |  |
| Lack of knowledge |  |  |  |  |  | 0.116 |
| No | 22 (21.0%) | 52 (31.1%) | 34 (21.3%) | 29 (19.9%) | 36 (22.6%) |  |
| Yes | 83 (79.0%) | 115 (68.9%) | 126 (78.8%) | 117 (80.1%) | 123 (77.4%) |  |
| Lack of time |  |  |  |  |  | 0.289 |
| No | 57 (54.3%) | 70 (41.9%) | 77 (48.1%) | 67 (45.9%) | 68 (42.8%) |  |
| Yes | 48 (45.7%) | 97 (58.1%) | 83 (51.9%) | 79 (54.1%) | 91 (57.2%) |  |
| Lack of motivation |  |  |  |  |  | 0.127 |
| No | 44 (41.9%) | 64 (38.3%) | 73 (45.6%) | 58 (39.7%) | 50 (31.4%) |  |
| Yes | 61 (58.1%) | 103 (61.7%) | 87 (54.4%) | 88 (60.3%) | 109 (68.6%) |  |
| Financial reasons |  |  |  |  |  | 0.449 |
| No | 74 (70.5%) | 125 (74.9%) | 118 (73.8%) | 100 (68.5%) | 106 (66.7%) |  |
| Yes | 31 (29.5%) | 42 (25.1%) | 42 (26.3%) | 46 (31.5%) | 53 (33.3%) |  |
| Difficulties with organizing |  |  |  |  |  |  |
| No | 73 (69.5%) | 115 (68.9%) | 106 (66.3%) | 83 (56.6%) | 90 (56.6%) |  |
| Yes | 32 (30.5%) | 52 (31.1%) | 54 (33.8%) | 63 (43.2%) | 69 (43.4%) |  |
| Health condition |  |  |  |  |  | 0.005 |
| No | 54 (51.4%) | 93 (55.7%) | 97 (60.6%) | 81 (55.5%) | 64 (40.3%) |  |
| Yes | 51 (48.6%) | 74 (44.3%) | 63 (39.4%) | 65 (44.5%) | 95 (59.7%) |  |
| Please identify the ‘modifiable risk factors’ for dementia from the below |  |  |  |  |  | 0.015 |
| Hypertension |  |  |  |  |  | 0.015 |
| No | 59 (56.2%) | 96 (57.5%) | 73 (45.6%) | 80 (54.8%) | 66 (41.5%) |  |
| Yes | 46 (43.8%) | 71 (42.5%) | 87 (54.4%) | 66 (45.2%) | 93 (58.5%) |  |
| Depression |  |  |  |  |  | 0.251 |
| No | 25 (23.8%) | 56 (33.5%) | 51 (31.9%) | 52 (35.6%) | 44 (27.7%) |  |
| Yes | 80 (76.2%) | 111 (66.5%) | 109 (68.1%) | 94 (64.4%) | 115 (72.3%) |  |
| Excessive alcohol consumption |  |  |  |  |  | 0.093 |
| No | 25 (23.8%) | 48 (28.7%) | 41 (25.6%) | 44 (30.1%) | 28 (17.6%) |  |
| Yes | 80 (76.2%) | 119 (71.3%) | 119 (74.4%) | 102 (69.9%) | 131 (74.8%) |  |
| Less education |  |  |  |  |  | 0.160 |
| No | 73 (69.5%) | 122 (73.1%) | 109 (68.1%) | 95 (65.1%) | 96 (60.4%) |  |
| Yes | 32 (30.5%) | 45 (26.9%) | 51 (31.9%) | 51 (34.9%) | 63 (39.6%) |  |
| Smoking |  |  |  |  |  | 0.095 |
| No | 35 (33.3%) | 69 (41.3%) | 52 (32.5%) | 53 (36.3%) | 43 (27.0%) |  |
| Yes | 70 (66.7%) | 98 (58.7%) | 108 (67.5%) | 93 (63.7%) | 116 (73.0%) |  |
| Head injury |  |  |  |  |  | 0.310 |
| No | 53 (50.5%) | 85 (50.9%) | 73 (45.6%) | 58 (39.7%) | 76 (47.8%) |  |
| Yes | 52 (49.5%) | 82 (49.1%) | 87 (54.4%) | 88 (60.3%) | 83 (52.2%) |  |
| Air pollution |  |  |  |  |  | 0.623 |
| No | 79 (75.2%) | 123 (73.7%) | 112 (70.0%) | 98 (67.1%) | 113 (71.1%) |  |
| Yes | 26 (24.8%) | 44 (26.3%) | 48 (30.0%) | 48 (32.9%) | 46 (28.9%) |  |
| Infrequent social contact |  |  |  |  |  | 0.206 |
| No | 60 (57.1%) | 90 (53.9%) | 83 (51.9%) | 76 (52.1%) | 69 (43.4%) |  |
| Yes | 45 (42.9%) | 77 (46.1%) | 77 (48.1%) | 70 (47.9%) | 90 (56.6%) |  |
| Diabetes |  |  |  |  |  | 0.701 |
| No | 55 (52.4%) | 86 (51.5%) | 81 (50.6%) | 81 (55.5%) | 75 (47.2%) |  |
| Yes | 50 (47.6%) | 81 (48.5%) | 79 (49.4%) | 65 (44.5%) | 84 (52.8%) |  |
| Obesity |  |  |  |  |  | 0.043 |
| No | 53 (50.5%) | 86 (51.5%) | 83 (51.9%) | 73 (50.0%) | 59 (37.1%) |  |
| Yes | 52 (49.5%) | 81 (48.5%) | 77 (48.1%) | 73 (50.0%) | 100 (62.9%) |  |
| Stroke |  |  |  |  |  | 0.885 |
| No | 60 (57.1%) | 88 (52.7%) | 85 (53.1%) | 74 (50.7%) | 82 (51.6%) |  |
| Yes | 45 (42.9%) | 79 (47.3%) | 75 (46.9%) | 72 (49.3%) | 77 (48.4%) |  |
| Hearing impairment |  |  |  |  |  | 0.030 |
| No | 82 (78.1%) | 127 (76.0%) | 110 (68.8%) | 115 (78.8%) | 104 (65.4%) |  |
| Yes | 23 (21.9%) | 40 (24.0%) | 50 (31.3%) | 31 (21.2%) | 55 (34.6%) |  |
| Physical inactivity |  |  |  |  |  | 0.013 |
| No | 41 (39.0%) | 52 (31.1%) | 68 (42.5%) | 53 (36.3%) | 40 (25.2%) |  |
| Yes | 64 (61.0%) | 115 (68.9%) | 92 (57.5%) | 93 (63.7%) | 119 (74.8%) |  |
| From where you would like to get the information regarding dementia risk factors? |  |  |  |  |  |  |
| Alzheimer’s disease associations |  |  |  |  |  | 0.001 |
| No | 83 (79.0%) | 129 (77.2%) | 126 (78.8%) | 113 (77.4%) | 98 (61.6%) |  |
| Yes | 22 (21.0%) | 38 (22.8%) | 34 (21.3%) | 33 (22.6%) | 61 (38.4%) |  |
| Internet |  |  |  |  |  | 0.508 |
| No | 26 (24.8%) | 54 (32.3%) | 54 (33.8%) | 43 (29.5%) | 44 (27.7%) |  |
| Yes | 79 (75.2%) | 113 (67.7%) | 106 (66.3%) | 103 (70.5%) | 115 (72.3%) |  |
| Healthy Aging website HMC |  |  |  |  |  | 0.006 |
| No | 49 (46.7%) | 102 (61.1%) | 108 (67.5%) | 86 (58.9%) | 83 (52.2%) |  |
| Yes | 56 (53.3%) | 65 (38.9%) | 52 (32.5) | 60 (41.1%) | 76 (47.8%) |  |
| Memory clinic training program HMC |  |  |  |  |  | 0.001 |
| No | 62 (59.0%) | 103 (61.7%) | 107 (66.9%) | 83 (56.8%) | 71 (44.7%) |  |
| Yes | 43 (41.0%) | 64 (38.3%) | 53 (33.1%) | 63 (43.2%) | 88 (55.3%) |  |
